# Predictive frameworks for youth basketball: a leakage-aware reassessment of athlete development and injury-risk models

**DOI:** 10.3389/fphys.2026.1912035

**Published:** 2026-10-22

**Authors:** Chen Yao

**Affiliations:** Nanjing Institute of Technology (NJIT), Nanjing, China

**Keywords:** agentic AI, basketball analytics, ethical AI, injury risk prediction, personalized training

## Abstract

**Introduction:**

Artificial intelligence is increasingly applied in sports analytics; however, exceptionally high predictive performance may result from target construction, data leakage, or deterministic label structures. This study reassessed predictive models for youth basketball using a leakage-aware framework.

**Methods:**

A publicly available youth-basketball dataset containing 1,000 records and 25 variables was analyzed. Development-score construction and injury-risk label structure were audited. Construction-related predictors were subsequently excluded, and model performance was evaluated using repeated cross-validation and a locked 200-athlete holdout. Subgroup analyses by gender and age and an external-validation feasibility assessment were also conducted.

**Results:**

Development_Score was reconstructed almost perfectly from its documented components, with repeated cross-validation R^2^ of 0.999925. Injury_Risk_Level also demonstrated deterministic structure based on strength and endurance. After excluding construction-related predictors, locked-holdout performance decreased to 0.750 accuracy, 0.329 balanced accuracy, 0.286 macro-F1, and 0.622 macro AUC. High-risk sensitivity was 0.000 across the overall sample and examined subgroups. No compatible public dataset was identified for external validation.

**Discussion:**

The initially exceptional performance largely reflected target construction and deterministic labeling rather than independent predictive information. These findings emphasize leakage-aware validation, transparent reporting, and cautious interpretation of sports-analytics prediction models.

## Introduction

Artificial intelligence (AI) is increasingly used in sport for performance analysis, training support, and health-related decision support (Chmait and Westerbeek, 2021; Munoz-Macho et al., 2024; Musat et al., 2024; Jianjun et al., 2025; Kim et al., 2025; Mateus et al., 2025; Xu and Baghaei, 2025). In basketball and other performance settings, machine-learning methods can integrate demographic, physical, technical, cognitive, and training variables to characterize athlete profiles and estimate performance- or risk-related outcomes (Chmait and Westerbeek, 2021; Munoz-Macho et al., 2024; Musat et al., 2024; Jianjun et al., 2025; Mateus et al., 2025; Xu and Baghaei, 2025). However, apparently strong predictive performance can be misleading when an outcome is directly constructed from predictor variables or when label definitions encode deterministic rules (Kaufman et al., 2012). Transparent reporting, data provenance, leakage-aware validation, class-sensitive performance metrics, and subgroup assessment are therefore necessary before predictive outputs are interpreted as actionable athlete-management evidence (Collins et al., 2024).

The sports-AI literature now spans athlete profiling, workload and health monitoring, performance prediction, and decision support, using methods that range from conventional regression and tree-based learning to deep-learning systems (Claudino et al., 2019; Chmait and Westerbeek, 2021; Van Eetvelde et al., 2021; Munoz-Macho et al., 2024; Musat et al., 2024; Jianjun et al., 2025; Mateus et al., 2025; Xu and Baghaei, 2025). Reviews of team-sport applications describe substantial methodological diversity and recurring interest in basketball, but they also emphasize that promising algorithmic performance does not by itself establish prospective usefulness (Claudino et al., 2019; Van Eetvelde et al., 2021). This distinction is especially important in athlete-health applications, where a model may rank individuals accurately in one dataset yet fail when target definitions, measurement processes, population characteristics, or class prevalence change. The value of a sports prediction model therefore depends on the credibility of its data-generating process and evaluation design as much as on the selected algorithm.

Three evaluation problems are particularly relevant to the present reassessment. First, target leakage can occur when the outcome is calculated from predictors or when a label encodes a deterministic rule, producing apparently exceptional performance without independent predictive information (Kaufman et al., 2012). Second, injury-risk data are commonly imbalanced, so accuracy or ROC-AUC can obscure failure in the minority group; class-sensitive measures and precision–recall analysis are needed to reveal whether high-risk cases are actually detected (He and Garcia, 2009; Saito and Rehmsmeier, 2015). Third, discrimination must be accompanied by calibration, transparent model specification, locked evaluation, and external validation before practical interpretation (Van Calster et al., 2019; Collins et al., 2024). These requirements motivate reporting that separates diagnostic reconstruction of the source labels from the residual prediction task that remains after construction-related variables are removed.

### Research gap

A central gap in applied sports analytics is not simply the availability of predictive algorithms, but the credibility of the evaluation connecting model output to real-world interpretation. Near-perfect performance can arise from target construction or deterministic label rules rather than from broadly generalizable predictive signal. The present paper therefore focuses on four linked issues raised during peer review: whether the development and injury-risk targets are independent of their predictors, whether performance remains after construction-related variables are removed, whether the evaluation is reproducible on a locked holdout with nested model selection, and whether subgroup behavior and external-validation feasibility support practical claims.

This gap is reinforced by evidence from sports-injury prediction research. A systematic review of 204 models found that most had high or unclear risk of bias, calibration was rarely reported, internal validation was incomplete, and none of the included studies externally validated a model (Bullock et al., 2022). The central challenge is therefore not to add algorithmic complexity to an uncertain target, but to demonstrate that a model is based on independently meaningful outcomes, evaluated without leakage, transparent about minority-class performance, and bounded by the evidence available for its intended population. The present study addresses this challenge through target reconstruction, proxy exclusion, a locked holdout, subgroup diagnostics, and an explicit external-validation feasibility audit.

### Motivation of the study

Basketball training involves simultaneous consideration of technical development, physical conditioning, and athlete health. The public dataset analyzed here provides a useful computational test bed, but its source documentation identifies youth athletes rather than a verified collegiate cohort and does not document the original recruiting institution or collection protocol. The motivation of this reassessment is therefore methodological: to determine what can be concluded from the available data without overstating provenance, prediction, fairness, or decision-support capability.

### Research objectives

The objective of this study is to reassess the original youth-basketball predictive framework using transparent, leakage-aware validation. Specifically, we audit how Development_Score and Injury_Risk_Level relate to their input features; quantify performance before and after removing construction-related injury predictors; document a locked holdout and nested model-selection procedure; evaluate gender and age subgroups using established disparity measures; assess whether a compatible independent public dataset permits external validation; and audit whether the previously described decision layer is genuinely optimized or is deterministic rule-based post-processing.

### Research questions

In pursuit of these objectives, the revised study addresses the following research questions:

RQ1. To what extent do the reported near-perfect development and injury-risk results reflect target construction or deterministic label structure rather than independent predictive signal?

RQ2. How does injury-risk performance change on a prespecified locked holdout after removing construction-related predictors, and how stable is performance across gender and age subgroups?

RQ3. What level of decision-support and external-validity claim is justified by the implemented workflow after auditing the former safety-weight and recommendation logic?

## Methods

This study was designed as a secondary, leakage-aware computational reassessment of a previously analyzed youth-basketball dataset. The objective of the revised methodology was not to preserve unusually high predictive performance, but to determine whether the two source-defined outcomes contained independent predictive information after explicitly auditing target construction, deterministic label structure, validation design, subgroup behavior, and the previously described recommendation layer. No new outcome definition, *post hoc* threshold search, or additional model-development branch was introduced after the reviewer-driven analysis was locked. The methodological approach follows established recommendations to prevent target leakage and to report prediction-model development and evaluation transparently (Kaufman et al., 2012; Collins et al., 2024).

### Data source, provenance, and scope

The analysis used the publicly available youth_basketball_training dataset hosted on Kaggle under the dataset identifier ziya07/youth-basketball-training (Ziya, n.d.) (Ziya, [[NoYear]]). The analyzed file contains 1,000 records and 25 columns representing youth-athlete demographic, physical, technical, cognitive/educational, training, and outcome variables. The observed age range in the analyzed file was 14–18 years. The exact CSV used for the revision was locked by SHA-256 checksum 29ff3b7012264dc987656843e3b3925bf6916c58a04f39ec3bd3bf3e1f95d807 so that the analytical source could be reproduced exactly.

The authors did not recruit participants, collect measurements, or modify the source records. Importantly, the public repository does not identify an originating institution, recruitment protocol, consent procedure, source-study ethics process, or a verifiable data-generation mechanism. It also does not establish whether individual rows represent observational measurements, simulated records, or another form of generated data. Accordingly, this manuscript treats the file as a public computational dataset and does not describe it as a prospectively collected or institutionally verified athlete cohort.

### Data integrity and preprocessing

Data-integrity checks were performed before modeling and included verification of file dimensions, duplicate-row screening, Athlete_ID uniqueness, outcome-class preservation, and consistency of the locked source file. Athlete_ID was excluded from all predictive modeling. Numeric predictors were retained in numeric form. Categorical predictors were one-hot encoded within the modeling pipeline, and continuous predictors were standardized only where required by the corresponding estimator. Preprocessing was performed within the modeling workflow so that transformation parameters were learned from training data rather than from the locked test set.

Two source-defined outcomes were retained. Development_Score was analyzed as a continuous endpoint. Injury_Risk_Level was retained as the original three-class outcome (Low, Moderate, and High); the Moderate class was not collapsed or discarded. A secondary High-versus-Rest formulation was used only for clinically interpretable discrimination, calibration, sensitivity, specificity, precision-recall, and subgroup diagnostics, not as a replacement for the primary three-class analysis.

### Development-score target-construction audit

Because the dataset documentation describes Development_Score as being calculated from strength, endurance, game performance, and academic performance, the first analysis treated this endpoint as a target-construction problem rather than assuming it was an independently observed outcome. Ordinary least-squares regression was used to reconstruct Development_Score from Strength, Endurance, Game_Performance_Index, and Academic_Performance_GPA. The reconstruction model was specified as:


$$\text{Development}\_{\text{Score}}_{\text{i}}={\text{β}}_{0}+{\text{β}}_{1}{\text{Strength}}_{\text{i}}+{\text{β}}_{2}{\text{Endurance}}_{\text{i}}+{\text{β}}_{3}{\text{GPI}}_{\text{i}}+{\text{β}}_{4}{\text{GPA}}_{\text{i}}+{\text{ϵ}}_{\text{i}}$$

Reconstruction stability was assessed with repeated cross-validation, and coefficient uncertainty was summarized with bootstrap 95% confidence intervals. The purpose of this audit was to test whether the near-perfect regression performance could be explained by direct mathematical dependence between the endpoint and its documented components, a recognized form of target leakage or target contamination (Kaufman et al., 2012).

A separate construction-excluded sensitivity analysis then removed Strength, Endurance, Game_Performance_Index, and Academic_Performance_GPA from the predictor matrix. Residual predictive information was assessed with leakage-safe regression models and a dummy mean-prediction baseline. The comparison included regularized linear and tree-based ensemble approaches already present in the locked analysis; model assessment used the same development/test separation described below.

### Injury-risk target-structure and leakage audit

Injury_Risk_Level was audited before substantive predictive interpretation. A depth-limited decision-tree procedure was used as a transparent rule-reconstruction test to determine whether a small number of predictors could reproduce the three-class labels. Tree depth was deliberately restricted so that any simple deterministic relationship would be visible rather than hidden within a complex ensemble. This diagnostic audit was not treated as evidence of real-world injury prediction.

The all-predictor classification workflow was retained only as a diagnostic reference. A prespecified construction-proxy-excluded sensitivity analysis then removed Strength and Endurance before model selection and final evaluation. All three outcome classes were preserved. The proxy-excluded analysis was designated as the principal injury-risk analysis for evaluating residual predictive information after the strongest label-construction relationships had been removed (**Table 1**).

**Table 1:** Locked-holdout injury-risk performance.

Feature set	Accuracy	Balanced accuracy	Macro-F1	Macro AUC	High AUC	High sensitivity	High specificity
All predictors — diagnostic	1.000	1.000	1.000	1.000	1.000	1.000	1.000
Construction-proxy-excluded	0.750	0.329	0.286	0.622	0.670	0.000	0.989

### Development/test split and nested model selection

A single locked 80:20 development/test split was fixed using random seed 20260810, yielding 800 development observations and 200 locked-test observations. The split was stratified to preserve the injury-risk class structure. The 200-observation test set was isolated before final model comparison and was not used for hyperparameter tuning, feature selection, threshold optimization, or model choice.

Candidate models were compared only within the 800-observation development set using repeated nested cross-validation. The archived injury-model evaluation contains 15 outer validation scores for each feature-set/model combination, while hyperparameter selection was confined to training folds within the nested procedure. This design separates model selection from final performance estimation and reduces optimistic bias from repeatedly evaluating candidate models on the same holdout.

For the construction-proxy-excluded injury analysis, XGBoost was the locked selected classifier. The retained specification was colsample_bytree = 0.8, learning_rate = 0.08, max_depth = 2, n_estimators = 400, and subsample = 0.8. XGBoost is a gradient-boosted decision-tree system that combines additive tree learning with regularization and efficient optimization (Chen and Guestrin, 2016). Once this model specification was fixed, the locked test set was used once for final performance estimation; subgroup analyses were performed from those same locked predictions rather than by refitting subgroup-specific models.

### Performance measures

Regression performance was summarized with the coefficient of determination (R²), mean absolute error (MAE), and root mean squared error (RMSE):


$${\text{R}}^{2}=1-[\Sigma_{\text{i}}{\left({\text{y}}_{\text{i}}-{\hat{\text{y}}}_{\text{i}}\right)}^{2}/\;\Sigma_{\text{i}}{\left({\text{y}}_{\text{i}}-\bar{\text{y}}\right)}^{2}]$$

$$\text{MAE}=(1/\text{n})\;\Sigma_{\text{i}}\;|{\text{y}}_{\text{i}}-{\hat{\text{y}}}_{\text{i}}|$$

$$\text{RMSE}=\surd [(1/\text{n})\;\Sigma_{\text{i}}\;{\left({\text{y}}_{\text{i}}-{\hat{\text{y}}}_{\text{i}}\right)}^{2}]$$

Three-class injury-risk performance was summarized using overall accuracy, balanced accuracy, macro-F1, macro one-versus-rest ROC-AUC, multiclass log loss, and multiclass Brier score. Because the outcome was imbalanced, class-sensitive metrics were emphasized rather than accuracy alone. Balanced accuracy and macro-F1 were defined as the unweighted mean across the K classes:


$$\text{Balanced accuracy}=(1/\text{K})\;\Sigma_{\text{k}}{\text{ Sensitivity}}_{\text{k}}$$

$$\text{Macro}-\text{F}1=(1/\text{K})\;\Sigma_{\text{k}}\text{ F}1_{\text{k}}$$

Probabilistic accuracy was evaluated with the multiclass Brier score, computed as the average squared difference between predicted class probabilities and observed class indicators:


$$\text{Brier}\_\text{multiclass}=(1/\text{n})\;\Sigma_{\text{i}}\;\Sigma_{\text{k}}\;{\left({\text{p}}_{\text{ik}}-1[{\text{y}}_{\text{i}}=\text{k}]\right)}^{2}$$

For the secondary High-versus-Rest analysis, discrimination and classification performance were summarized with ROC-AUC, average precision, sensitivity, specificity, and the binary Brier score:


$$\text{Brier}\_\text{binary}=(1/\text{n})\;\Sigma_{\text{i}}\;{\left({\text{p}}_{\text{i}}-{\text{y}}_{\text{i}}\right)}^{2}$$

Precision-recall performance was reported because it can be more informative than ROC summaries when the positive class is uncommon (Saito and Rehmsmeier, 2015). Calibration was assessed separately from discrimination because a model can rank athletes while still assigning poorly calibrated probabilities (Van Calster et al., 2019). No *post hoc* threshold search was performed on the locked test set.

### External-validation feasibility audit

External validation was treated as direct evaluation of the locked model in an independent dataset without changing the target definition, predictor meaning, or model specification. Candidate public datasets were screened against prespecified eligibility criteria: (1) publicly accessible individual-level data; (2) a compatible youth-basketball population; (3) an observed, semantically compatible injury-risk outcome; (4) availability of sufficient predictors required by the locked model; and (5) the ability to apply the model without outcome relabeling, refitting, or redefining the prediction problem. The outcome of this feasibility audit is reported in the Results. Incompatible datasets were not repurposed to manufacture a nominal external-validation analysis.

### Subgroup and fairness assessment

Subgroup analyses used the same locked-test predictions and were descriptive rather than model-refitting exercises. Performance was summarized by gender and by age strata of 14–15 and 16–18 years, matching the observed age range of the analyzed file. For High-versus-Rest classification, subgroup sample size, High-risk count, balanced accuracy, macro-F1, sensitivity, and specificity were reported. Wilson 95% confidence intervals were used for subgroup sensitivity and specificity because Wilson intervals have better coverage properties than the simple Wald interval for binomial proportions, especially with small event counts (Newcombe, 1998).

Fairness-related diagnostics were based on established group-conditional error-rate concepts (Hardt et al., 2016). For group A = a, the selection rate, true-positive rate, and false-positive rate were defined as:


$${\text{SR}}_{\text{a}}=\text{P}(\hat{\text{Y}}=\;1|\text{A}=\text{a})$$

$${\text{TPR}}_{\text{a}}=\text{P}(\hat{\text{Y}}=1|\text{Y}=1,\text{ A}=\text{a})$$

$${\text{FPR}}_{\text{a}}=\text{P}(\hat{\text{Y}}=1|\text{Y}=0,\text{ A}=\text{a})$$

For two-group comparisons, demographic-parity difference was the absolute difference in selection rates, equal-opportunity difference was the absolute difference in true-positive rates, and equalized-odds difference was the larger of the absolute true-positive-rate and false-positive-rate differences:


$$\text{DPD}=|{\text{SR}}_{\text{a}}-\text{SR}\_\text{b}|$$

$$\text{EOD}=|{\text{TPR}}_{\text{a}}-\text{TPR}\_\text{b}|$$

$$\text{Equalized}-\text{odds difference}=\text{max}(|{\text{TPR}}_{\text{a}}-\text{TPR}\_\text{b}|,\;|{\text{FPR}}_{\text{a}}-\text{FPR}\_\text{b}|)$$

These quantities were used as transparency and failure-mode diagnostics rather than as a certification of algorithmic fairness. Their interpretation was conditioned on absolute subgroup performance, particularly sensitivity for the High-risk class (**Figure 1**).

**Figure 1:**
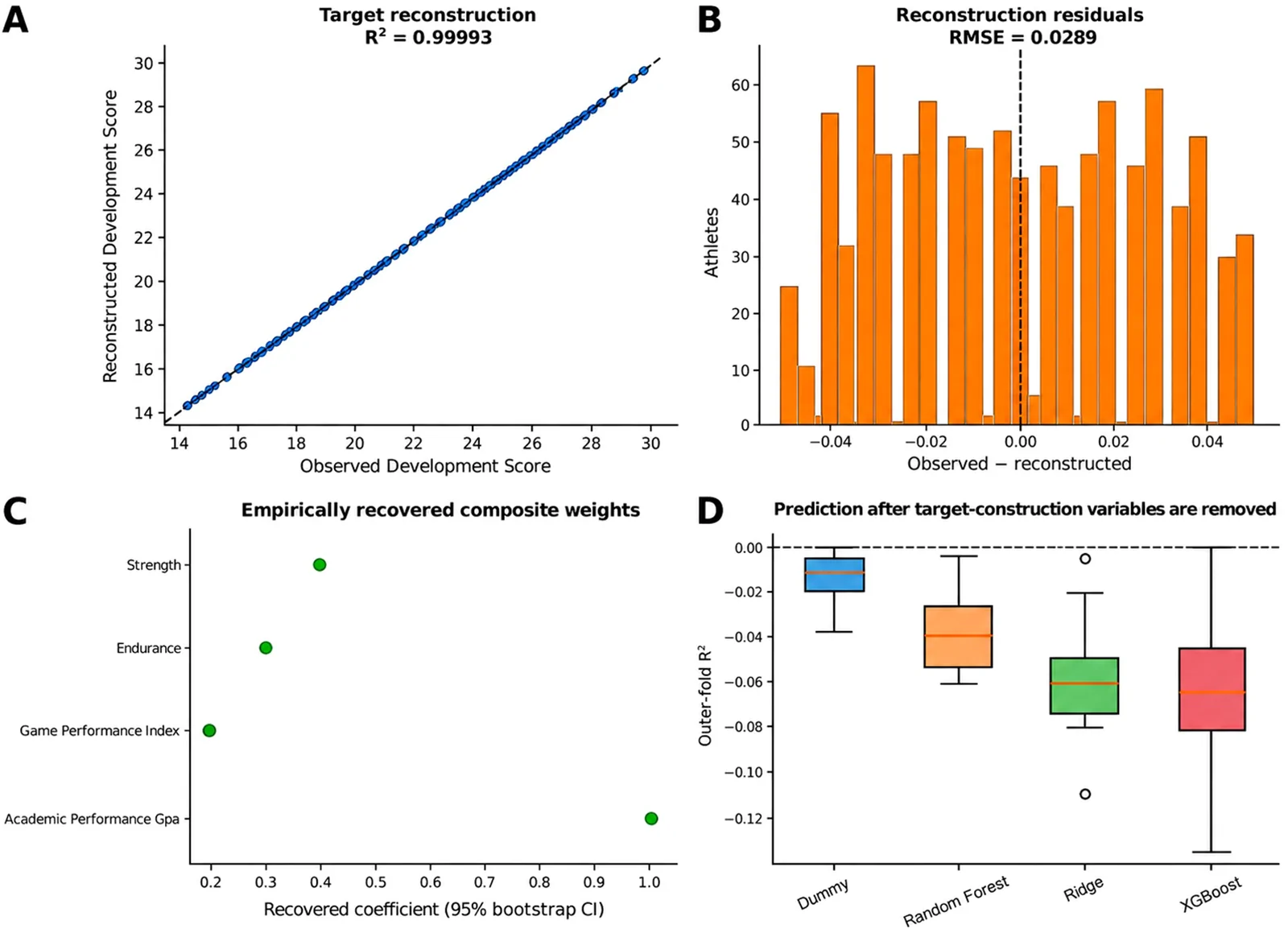
Development-score construction audit. **(A)** Reconstructed versus observed development scores. **(B)** Distribution of reconstruction residuals. **(C)** Empirically recovered composite weights. **(D)** Outer-fold R² values after removing target-construction variables.

### Decision-support implementation audit

The earlier manuscript described a safety-weighted utility and characterized the downstream recommendation logic as policy optimization. The revised analysis audited the implemented code path rather than accepting that terminology. For athlete i and safety weight λ, the archived utility expression was:


$${\text{U}}_{\text{i}}(\text{λ})={\hat{\text{D}}}_{\text{i}}-{\text{λ}\hat{\text{p}}}_{\text{i}},\text{High}$$

where ${\hat{\text{D}}}_{\text{i}}$ is predicted development and ${\hat{\text{p}}}_{\text{i}}$, High is the predicted probability of High injury risk. The audit examined the stored/reconstructed λ sweep across the original 0–10 range and evaluated whether changing λ altered predicted development, predicted risk, recommendation assignment, or only the arithmetic value of _i_(λ). The recommendation rules were inspected directly to determine whether λ was an input to action selection. Because the implemented system contained no state-transition model, environment interaction, trial-and-error learning, online adaptation, or learned sequential policy, the revised methodology does not describe it as reinforcement learning, a Markov decision process, or agentic AI. Any retained recommendation logic is treated strictly as deterministic rule-based post-processing.

### Reproducibility and ethical scope

All reviewer-driven analyses used the fixed random seed 20260810. The reproducibility package is intended to preserve the exact source checksum, split definition, fold assignments, nested-validation summaries, selected model specification, row-level locked-test predictions, subgroup tables, external-validation screening log, fitted model, figures, and executable code. Methods and results are deliberately separated: numerical model performance, recovered coefficients, target-reconstruction accuracy, subgroup outcomes, and external-validation findings are reported in the Results rather than embedded in this section.

This work involved secondary computational analysis of a publicly available dataset. The authors had no participant contact and used no private identifiable information. Because the original data-generation process and source-study ethics are not documented by the public repository, this study makes no claim about the ethics approval, consent process, or observational authenticity of the original records.

## Results

The reviewer-driven reanalysis changed the interpretation of the original results. The near-perfect Development_Score and all-predictor injury results were reproducible, but both were explained by target construction or deterministic label structure. The principal evidence therefore comes from leakage-aware sensitivity analyses, locked-holdout performance, external-validation feasibility, and subgroup diagnostics.

### Development-score construction

Development_Score was reconstructed almost exactly from the four components documented by the public source. The recovered linear equation yielded in-sample R² = 0.999926 and repeated cross-validation R² = 0.999925 ± 0.000002, with RMSE = 0.0290. Bootstrap coefficient intervals were narrow: Strength 0.399735 (95% CI 0.399077–0.400385), Endurance 0.300373 (0.299809–0.300989), Game_Performance_Index 0.199996 (0.199874–0.200120), and Academic_Performance_GPA 1.001468 (0.997310–1.005477) (**Table 2**). After these construction variables were removed, the predictive task collapsed to approximately null performance; for example, the leakage-safe XGBoost repeated-validation mean R² was -0.0616, while the dummy comparator was -0.0119. On the locked holdout, the leakage-safe dummy model produced R² = -0.0001, MAE = 2.8970, and RMSE = 3.4297. Thus, the original R²≈1 result is interpreted as reconstruction of a source-defined composite rather than evidence of exceptional independent prediction. The coefficient estimates and bootstrap intervals are visualized in **Supplementary Figure S1**.

**Table 2:** Target-construction and leakage audit summary.

Outcome	Audit	Result	Interpretation
Development_Score	Documented component reconstruction	Repeated-CV R² = 0.999925 ± 0.000002; RMSE = 0.0290	Near-perfect performance reflects source-defined composite construction.
Development_Score	Construction variables removed	XGBoost mean R² = -0.0616; dummy mean R² = -0.0119	No useful independent signal remained in the leakage-safe task.
Injury_Risk_Level	Class composition	Low 762; Moderate 131; High 107	All three source classes were retained.
Injury_Risk_Level	Depth-limited rule audit	Depth-4 macro-F1 = 1.00 using Strength and Endurance	Labels are deterministically structured by construction-related predictors.

### Injury-risk target structure and corrected validation

The three-class injury target could be reconstructed perfectly by a shallow decision tree using Strength and Endurance (**Figure 2**). Consistent with this finding, the all-predictor XGBoost model achieved perfect nested-validation and locked-holdout discrimination. These results are therefore reported as diagnostic evidence of target determinism, not as proof of real-world injury prediction capability.

**Figure 2:**
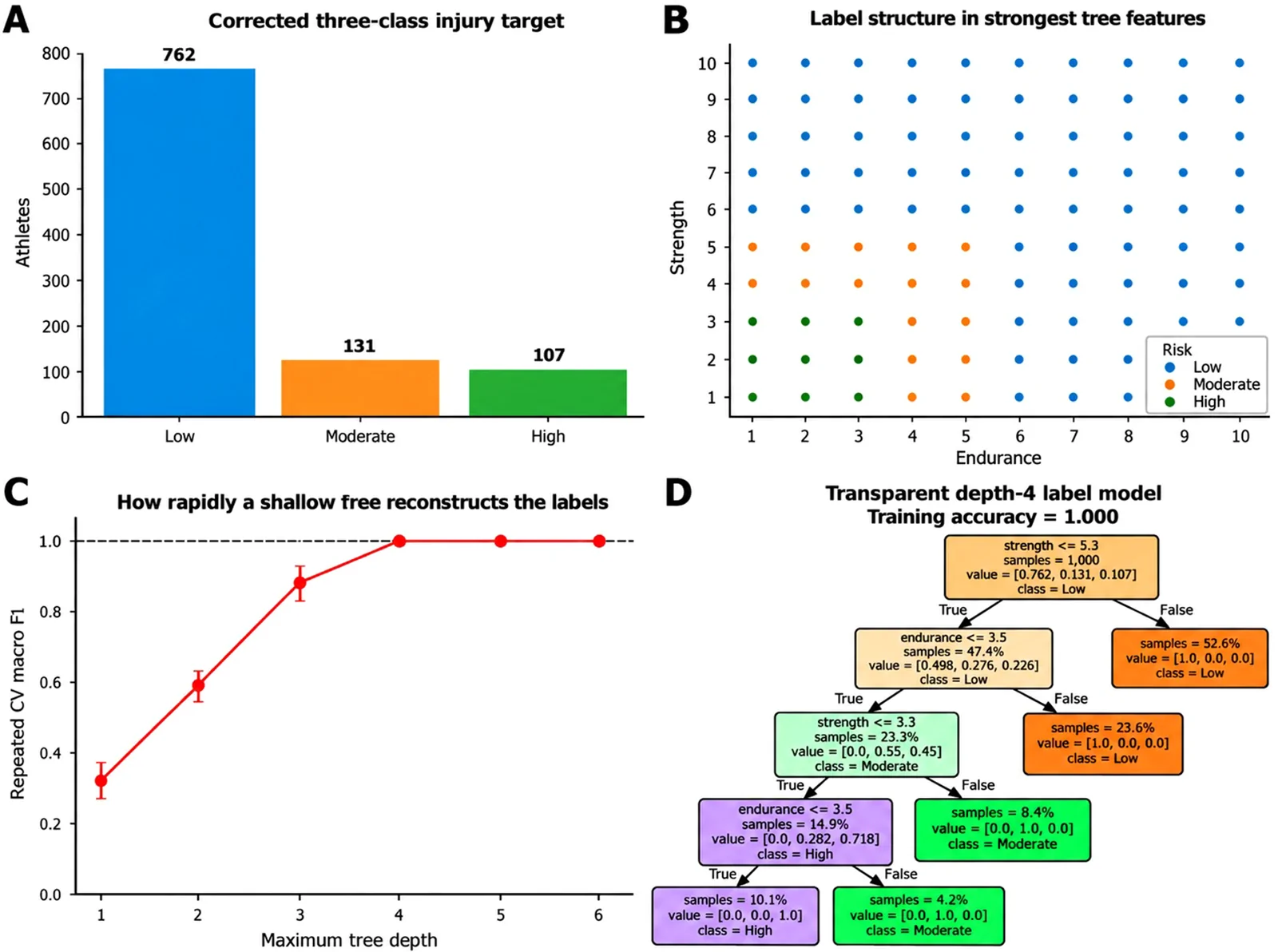
Injury-risk target audit. **(A)** Source-class composition. **(B)** Relationship of Strength and Endurance to injury-risk labels. **(C)** Depth-dependent rule reconstruction. **(D)** Shallow decision-tree representation used to diagnose deterministic target structure.

After Strength and Endurance were excluded, XGBoost performance on the locked 200-athlete holdout fell to accuracy 0.750, balanced accuracy 0.329, macro-F1 0.286, macro one-vs-rest AUC 0.622, and multiclass Brier score 0.398. For High-risk versus Rest, AUC was 0.670, average precision 0.218, Brier score 0.098, sensitivity 0.000, and specificity 0.989 (**Figure 4**). The confusion matrix contained 150 correctly predicted Low cases, 26 Moderate cases predicted as Low, 22 High cases predicted as Low, and two Low cases predicted as High; no Moderate or High case was correctly classified. The corrected results therefore do not support reliable High-risk detection. A direct comparison of the diagnostic and proxy-excluded locked metrics is provided in **Supplementary Figure S2**; **Figure 3**.

**Figure 3:**
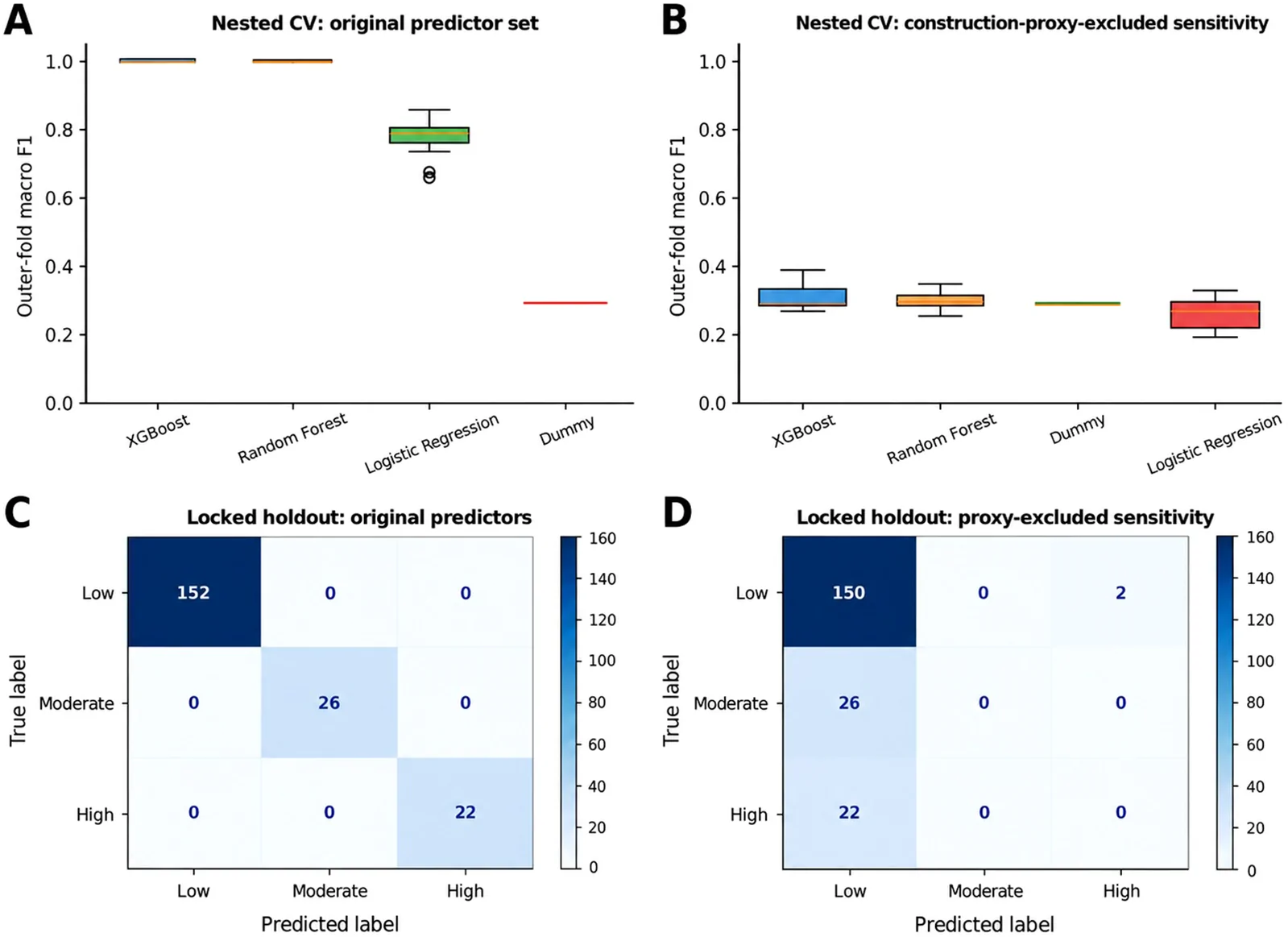
Corrected internal validation. **(A)** Outer-fold macro-F1 scores using the original predictor set. **(B)** Outer-fold macro-F1 scores after construction-proxy exclusion. **(C)** Locked-holdout confusion matrix using original predictors. **(D)** Locked-holdout confusion matrix after construction-proxy exclusion.

**Figure 4:**
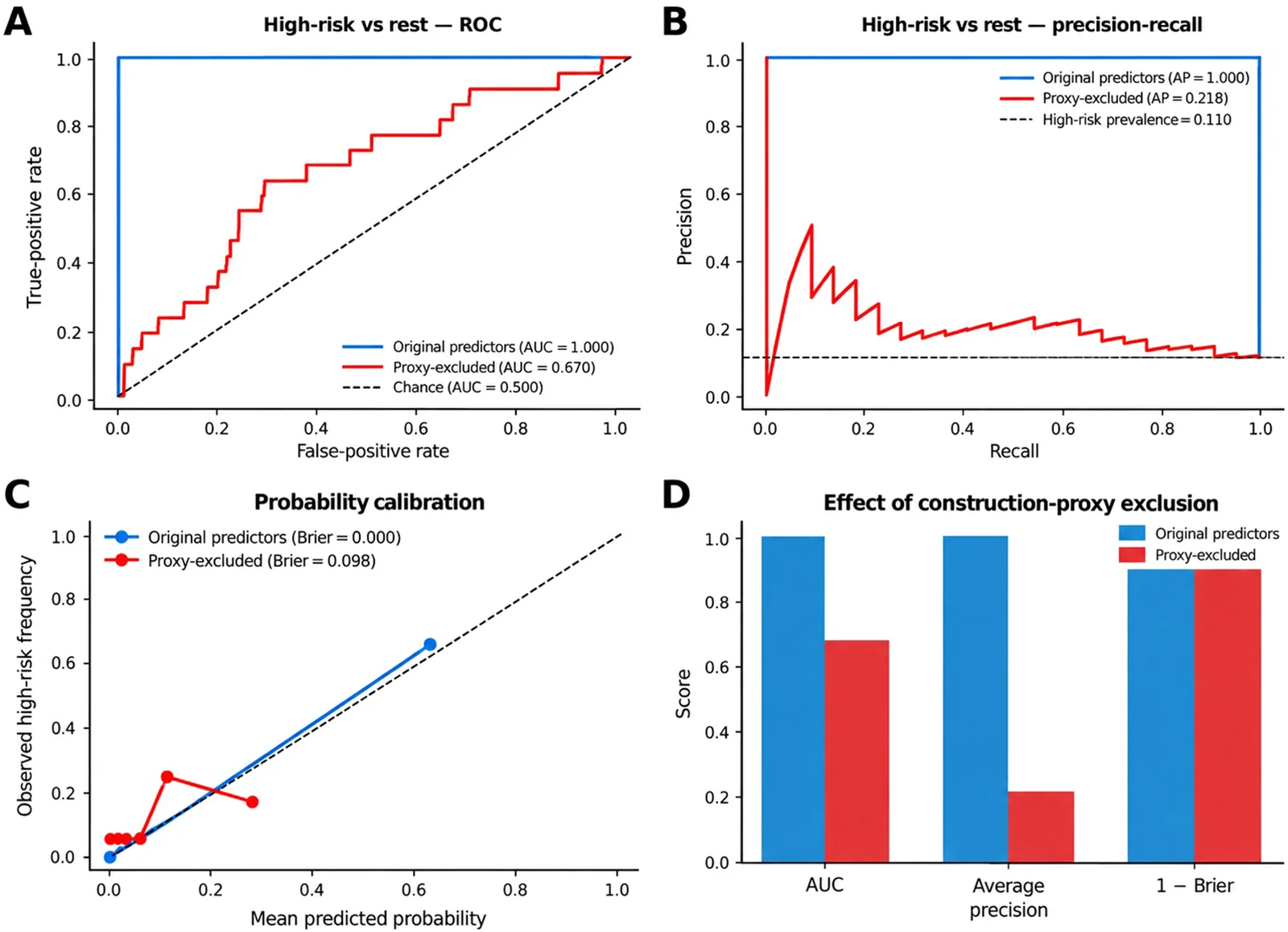
Audit of the former perfect injury-risk performance. **(A)** High-risk ROC curves. **(B)** High-risk precision–recall curves. **(C)** Probability calibration. **(D)** Comparison of AUC, average precision, and 1−Brier score between the diagnostic all-predictor and construction-proxy-excluded models.

### External validation and subgroup/fairness audit

The external-validation feasibility audit did not identify a public individual-level dataset that could evaluate the locked model without changing the population, target meaning, predictor definitions, or model specification. Available candidate datasets instead described different populations or outcomes such as injury occurrence, injury severity, rehabilitation status, or emergency-department diagnoses. External validation was therefore not performed; this is a limitation rather than a substituted validation claim.

Within the verified locked holdout, High-risk sensitivity was zero in females, males, ages 13–15, and ages 16–18 (**Table 3**). Gender demographic-parity difference was 0.0008, equal-opportunity difference 0.0000, false-positive-rate difference 0.0005, and equalized-odds difference 0.0005. Corresponding age-group differences were 0.0152, 0.0000, 0.0172, and 0.0172. These small disparity values do not demonstrate fairness: they arise in a degenerate setting in which no true High-risk athlete was detected in any subgroup. The subgroup analysis is therefore interpreted as a transparency and failure-mode audit. Subgroup performance and the formal disparity measures are visualized in **Supplementary Figures S3** and **S4**.

**Table 3:** Locked-holdout subgroup performance for the construction-proxy-excluded model.

Variable	Subgroup	N	High N	Balanced accuracy	Macro-F1	High sensitivity (95% CI)	High specificity
Gender	Female	104	13	0.329	0.279	0.000 (0.000–0.228)	0.989
Gender	Male	96	9	0.329	0.292	0.000 (0.000–0.299)	0.989
Age	13–15	68	6	0.333	0.298	0.000 (0.000–0.390)	1.000
Age	16–18	132	16	0.326	0.279	0.000 (0.000–0.194)	0.983

### Decision-support implementation audit

Across the former λ sweep from 0 to 10, mean predicted development remained 22.1261 and mean predicted High-risk probability remained 0.131000. Increasing λ therefore reduced the arithmetic utility with slope -0.131000 per unit λ but changed neither prediction nor recommendation behavior. Because λ was not used by the deterministic recommendation rule, λ = 3 was not an empirically selected operating point and the former reward-risk frontier did not represent optimization of actions. The associated utility histogram, policy frontier, utility-ranked athlete list, and automated recovery-triage interpretation were removed from the revised evidentiary chain. The arithmetic utility decline and invariance of predictions and recommendations are shown in **Supplementary Figure S5**.

## Discussion

### Addressing research questions

The revised analyses change the interpretation of each research question. For RQ1, the near-perfect Development_Score result is explained by direct construction of the outcome from Strength, Endurance, Game_Performance_Index, and Academic_Performance_GPA. Similarly, the original perfect injury-risk classification is reproduced by a shallow rule based on Strength and Endurance. These findings make the original perfect metrics useful as diagnostics of target structure, but inappropriate as evidence of exceptional independent prediction. For RQ2, removing the construction-related injury predictors produced a model with substantially weaker balanced accuracy and macro-F1 and, critically, zero High-risk sensitivity on the locked holdout. The same failure was observed across all examined gender and age subgroups. For RQ3, the implementation audit showed that the former λ parameter did not modify actions and that recommendations were deterministic rules rather than a learned or optimized policy. The defensible interpretation is therefore a reproducible supervised-learning sensitivity analysis with transparent rule-based post-processing, not an autonomous or reinforcement-learning system.

### Comparison with prior sports-AI literature

The results provide a counterpoint to literature describing the potential of artificial intelligence for athlete performance and injury-risk modeling. Those reviews show that neural networks, support-vector machines, decision trees, ensemble methods, and other machine-learning approaches have been widely explored in team sports, including basketball (Claudino et al., 2019). However, they also call for prospective evaluation and stronger evidence of practical predictive performance (Claudino et al., 2019; Van Eetvelde et al., 2021). In the present data, algorithmic sophistication did not resolve the central problem: the strongest results arose because the targets were constructed from, or deterministically related to, the predictors. The important contribution is therefore the identification of why performance was perfect and the measurement of what remained after those relationships were removed.

The deterioration observed after proxy exclusion is consistent with broader methodological concerns in sports-injury prediction. Bullock et al. (2022) reported that 98% of 204 reviewed models had high or unclear risk of bias, only 47% of studies used internal validation, calibration was reported in only 7%, and none externally validated a model (Bullock et al., 2022). Our locked analysis does not reproduce every limitation identified in that review, but it illustrates the same general warning: high headline discrimination can coexist with weak evidence for generalizable or actionable prediction. The use of XGBoost (Chen and Guestrin, 2016) provided a flexible nonlinear learner, yet no algorithm can correct an outcome whose apparent predictability is dominated by target construction. This is why the all-predictor model is retained only as a diagnostic audit and the proxy-excluded model determines the practical interpretation.

### Theoretical and practical implications

The principal implication is methodological. Sports-analytics studies can report excellent discrimination or fit while still offering little independent predictive information if the target is calculated from inputs or labels follow deterministic rules. In the present dataset, overall accuracy of 0.750 after proxy exclusion might appear acceptable in isolation, yet balanced accuracy of 0.329, macro-F1 of 0.286, and zero High-risk sensitivity reveal severe failure on clinically or operationally important minority classes. This illustrates why class-sensitive metrics, locked evaluation, and target-definition audits are necessary alongside headline accuracy or AUC. Practically, the current model should not be used to automate injury-prevention, recovery, or training-intensity decisions. Future athlete-management applications require independently collected outcomes, prospectively defined predictors, external validation, and evaluation of calibration and subgroup performance before deployment.

The metric pattern further explains why overall accuracy was insufficient. Low-risk observations formed 76.2% of the source data, and the proxy-excluded model predicted nearly every locked-test observation as Low. Under this failure mode, accuracy remained 0.750 even though balanced accuracy was 0.329, macro-F1 was 0.286, and High-risk sensitivity was 0.000. This agrees with established guidance that imbalanced classification requires class-sensitive evaluation and that precision–recall summaries can be more informative than ROC summaries when the positive class is uncommon (He and Garcia, 2009; Saito and Rehmsmeier, 2015). The High-risk average precision of 0.218 and Brier score of 0.098 should therefore be interpreted alongside the zero-sensitivity operating point, rather than as substitutes for it.

Calibration also remains central to any future version of the work. A model can discriminate between higher- and lower-risk individuals while assigning probabilities that are systematically too high or too low; calibration must therefore be examined separately from ranking performance (Van Calster et al., 2019). TRIPOD+AI likewise emphasizes transparent specification of predictors, outcomes, validation, performance measures, and data limitations (Collins et al., 2024). In this study, the calibration and subgroup analyses do not rescue the classifier: they make its failure visible. The correct practical response is not *post hoc* threshold tuning on the locked test set, but development of a new model using independently observed outcomes and prospectively defined predictors, followed by internal and external validation.

### Novelty of study

The contribution of the study is not a new agentic or reinforcement-learning architecture. Its novelty lies in a reviewer-driven forensic reassessment that connects public-data provenance, empirical target reconstruction, deterministic-label diagnosis, construction-proxy exclusion, locked-holdout reproducibility, external-validation feasibility, and subgroup/fairness auditing within one sports-analytics case study. This redesign converts initially impressive but potentially misleading performance into a transparent account of what the dataset can and cannot support.

This contribution is deliberately different from claiming a novel learning architecture. The study demonstrates an auditable sequence in which data provenance is checked, target construction is empirically reconstructed, leakage-related predictors are separated from residual predictors, model selection is confined to development data, performance is locked before subgroup analysis, and unsupported decision-system claims are removed. That sequence operationalizes established concerns about leakage and transparent prediction-model reporting (Collins et al., 2024; Kaufman et al., 2012) within a sports-analytics example. It also shows that a negative or weak corrected result can be scientifically informative when it prevents a deterministic label rule from being misrepresented as deployable athlete-risk intelligence.

### Limitations

Several limitations define the scope of inference. First, the analysis relies on a public Kaggle dataset whose repository documents the variables and target descriptions but does not provide sufficient information to independently verify the originating institution, recruitment procedure, consent process, authenticity of each athlete record, or whether records were observational or simulated. Second, both target definitions are source-defined; Development_Score is explicitly constructed from predictor variables, and Injury_Risk_Level exhibits deterministic structure involving Strength and Endurance. Third, after those injury construction proxies were removed, the model failed to detect any High-risk athlete in the locked holdout, so the current classifier is not suitable for safety-critical use. Fourth, subgroup fairness metrics are constrained by this degenerate High-risk performance and cannot establish fairness. Fifth, the feasibility audit found no compatible public participant-level dataset for direct external validation without changing the problem definition; its candidate-screening log will be supplied in the public archive. Finally, the deterministic recommendation rules were not prospectively tested, did not adapt online, and were not linked to observed post-recommendation outcomes; no claim of clinical or coaching effectiveness is therefore made.

The absence of external validation is particularly important. The feasibility audit used strict compatibility criteria because relabeling a different outcome, refitting the model, or changing the predictor set would constitute a new study rather than validation of the locked model. This decision is conservative but methodologically necessary. Until an independently collected youth-basketball dataset contains semantically compatible injury-risk outcomes and sufficient predictors, transportability cannot be estimated. Similarly, the subgroup findings are descriptive failure-mode evidence from one locked sample; they neither establish equal performance nor support claims of fairness. Future work should prespecify subgroup hypotheses, recruit adequate numbers of minority-class cases, evaluate calibration within relevant groups, and test whether predictions improve decisions or outcomes in a prospective setting.

## Conclusion

This reviewer-driven reassessment demonstrates that the strongest results in the original youth-basketball analysis were dominated by target construction rather than by broadly generalizable predictive learning. Development_Score was reconstructed almost exactly from its documented components, and Injury_Risk_Level was deterministically recoverable from Strength and Endurance. Once these injury construction proxies were excluded, locked-holdout performance fell markedly and High-risk sensitivity was zero, including across all examined gender and age subgroups. No compatible public dataset was identified for strict external validation. Re-audit also showed that the former safety-weight parameter changed only arithmetic utility and did not generate the deterministic recommendations; consequently, claims of agentic AI, reinforcement learning, policy optimization, and validated automated recovery triage are not supported. The resulting contribution is a transparent, leakage-aware sports-analytics case study that emphasizes target auditing, reproducible validation, subgroup failure analysis, and appropriately bounded claims as prerequisites for future athlete decision-support research.

## Data Availability

The original contributions presented in the study are included in the article/**Supplementary Material**. Further inquiries can be directed to the corresponding author.

## Supplementary material

Supplementary Figure 1 Development-score reconstruction coefficients. Points show recovered ordinary least-squares coefficients and horizontal bars show available bootstrap 95% confidence intervals. The intercept estimate was −0.003831; its confidence interval was not available in the embedded outputs.

Supplementary Figure 2 Locked injury-risk metric comparison. The diagnostic all-predictor model is contrasted with the construction-proxy-excluded model across accuracy, balanced accuracy, macro-F1, macro one-vs-rest AUC, High-risk AUC, average precision, sensitivity, and specificity.

Supplementary Figure 3 Locked-test subgroup failure-mode audit. Panel A compares balanced accuracy, macro-F1, and High-risk specificity across gender and age groups. Panel B shows zero High-risk sensitivity with Wilson 95% confidence intervals in every subgroup.

Supplementary Figure 4 High-risk-versus-Rest disparity metrics. Gender and age-group demographic-parity, equal-opportunity, false-positive-rate, and equalized-odds differences are shown. Small values are not evidence of fairness because High-risk sensitivity was zero in every subgroup.

Supplementary Figure 5 Implementation audit of the former λ sweep. Panel A shows the linear arithmetic decline in mean utility. Panels B and C show that predicted development, predicted High-risk probability, and recommendation behavior were invariant across λ = 0–10.

Supplementary Figure 6 Leakage-aware revision and interpretation workflow. The diagram summarizes source auditing, target reconstruction, construction-variable exclusion, locked evaluation, subgroup and calibration diagnostics, implementation auditing, and the resulting bounded interpretation.

Supplementary Table 1 Data provenance, integrity checks, and analytic outcomes.

Supplementary Table 2 Preprocessing, splitting, and locked-model specification.

Supplementary Table 3 Development_Score reconstruction coefficients. Reconstruction equation: Development_Score = −0.003831 + 0.399735(Strength) + 0.300373(Endurance) + 0.199996(Game_Performance_Index) + 1.001468(Academic_Performance_GPA).

Supplementary Table 4 Development_Score construction and proxy-excluded performance.

Supplementary Table 5 Locked injury-risk performance.

Supplementary Table 6 Construction-proxy-excluded locked-test confusion matrix.

Supplementary Table 7 Locked-test subgroup performance.

Supplementary Table 8 High-risk-versus-Rest disparity metrics.

Supplementary Table 9 Strict external-validation feasibility criteria.

Supplementary Table 10 Reconstructed λ implementation audit.The utility decreases linearly by 0.131000 per unit increase in λ. Because predictions and recommendations remain unchanged, this sweep is an arithmetic sensitivity check and not an optimized policy frontier.

Supplementary Table 11 Planned public reproducibility-archive manifest.
