# Supplementary material for: Predictive frameworks for youth basketball: a leakage-aware reassessment of athlete development and injury-risk models
Source: Front Physiol. 2026 Oct 22;17:1912035. doi: 10.3389/fphys.2026.1912035 (PMC13736777; doi:10.3389/fphys.2026.1912035)
Supplement: Supplementary file 1 [file SupplementaryFile1.docx]

Supplementary Material

This in-document supplement consolidates all aggregate supporting data that can be verified from the locked revision outputs available with the manuscript. It does not substitute for the planned public reproducibility archive. Row-level locked-test predictions, exact fold assignments, the complete external-validation candidate-screening log, fitted model objects, and executable code are repository artifacts and are not reproduced or inferred here because those files were not embedded in the manuscript package.

All values below use the fixed analysis seed 20260810 and the same definitions reported in the main manuscript. The diagnostic all-predictor results are retained solely to show target structure; the construction-proxy-excluded analysis is the relevant sensitivity analysis.

Supplementary Table S1. Data provenance, integrity checks, and analytic outcomes

| Item | Verified value | Interpretive note |
| --- | --- | --- |
| Public source | Kaggle: ziya07/youth-basketball-training | CC0: Public Domain |
| Exact-file checksum | SHA-256 29ff3b7012264dc987656843e3b3925bf6916c58a04f39ec3bd3bf3e1f95d807 | Locks the CSV version analyzed |
| Dataset dimensions | 1,000 records; 25 columns | Youth-athlete records as described by the public repository |
| Duplicate audit | 0 exact duplicate rows; 0 duplicate Athlete_ID values | Athlete_ID excluded from prediction |
| Continuous outcome | Development_Score | Source-defined composite endpoint |
| Three-class outcome | Injury_Risk_Level | Low 762; Moderate 131; High 107; no class removed |
| Provenance limitation | Originating institution, recruitment, consent, source ethics, and observational/simulated status undocumented | Record authenticity and collection context cannot be independently verified |

Supplementary Table S2. Preprocessing, splitting, and locked-model specification

| Component | Specification |
| --- | --- |
| Numeric predictors | Retained as numeric; standardized where required by the model pipeline |
| Categorical predictors | One-hot encoded within the modeling pipeline |
| Identifier | Athlete_ID excluded from prediction |
| Locked split | Stratified 80:20 split: 800 development observations and 200 locked-test observations |
| Random seed | 20260810 |
| Selection rule | Candidate comparison and hyperparameter selection confined to development-data training folds; locked test not used for selection |
| Construction-proxy exclusion | Strength and Endurance removed for the injury-risk sensitivity analysis |
| Selected classifier | XGBoost: colsample_bytree 0.8; learning_rate 0.08; max_depth 2; n_estimators 400; subsample 0.8 |

Supplementary Table S3. Development_Score reconstruction coefficients

| Term | Estimate | Bootstrap 95% CI |
| --- | --- | --- |
| Intercept | −0.003831 | Not available in the embedded outputs |
| Strength | 0.399735 | 0.399077–0.400385 |
| Endurance | 0.300373 | 0.299809–0.300989 |
| Game_Performance_Index | 0.199996 | 0.199874–0.200120 |
| Academic_Performance_GPA | 1.001468 | 0.997310–1.005477 |

Reconstruction equation: Development_Score = −0.003831 + 0.399735(Strength) + 0.300373(Endurance) + 0.199996(Game_Performance_Index) + 1.001468(Academic_Performance_GPA).

Supplementary Table S4. Development_Score construction and proxy-excluded performance

| Analysis | R² | MAE | RMSE | Interpretation |
| --- | --- | --- | --- | --- |
| Four-component OLS, in sample | 0.999926 | Not reported | Not reported | Near-exact source-defined reconstruction |
| Four-component OLS, repeated CV | 0.999925 ± 0.000002 | 0.0250 | 0.0290 | Construction result reproduced across resamples |
| Construction variables removed, XGBoost repeated validation | −0.0616 mean | Not available | Not available | No useful independent signal |
| Construction variables removed, dummy repeated validation | −0.0119 mean | Not available | Not available | Comparator also approximately null |
| Construction variables removed, locked dummy | −0.0001 | 2.8970 | 3.4297 | Locked baseline performance |

Supplementary Table S5. Locked injury-risk performance

| Metric | All predictors—diagnostic | Construction-proxy-excluded |
| --- | --- | --- |
| Accuracy | 1.000 | 0.750 |
| Balanced accuracy | 1.000 | 0.329 |
| Macro-F1 | 1.000 | 0.286 |
| Macro one-vs-rest AUC | 1.000 | 0.622 |
| Multiclass Brier score | 0.000 | 0.398 |
| High-vs-Rest AUC | 1.000 | 0.670 |
| High-vs-Rest average precision | 1.000 | 0.218 |
| High-vs-Rest Brier score | 0.000 | 0.098 |
| High-risk sensitivity | 1.000 | 0.000 |
| High-risk specificity | 1.000 | 0.989 |

Supplementary Table S6. Construction-proxy-excluded locked-test confusion matrix

| True class | Predicted Low | Predicted Moderate | Predicted High | Row total |
| --- | --- | --- | --- | --- |
| Low | 150 | 0 | 2 | 152 |
| Moderate | 26 | 0 | 0 | 26 |
| High | 22 | 0 | 0 | 22 |
| Column total | 198 | 0 | 2 | 200 |

Supplementary Table S7. Locked-test subgroup performance

| Variable | Subgroup | N | High N | Balanced accuracy | Macro-F1 | High sensitivity (95% CI) | High specificity |
| --- | --- | --- | --- | --- | --- | --- | --- |
| Gender | Female | 104 | 13 | 0.329 | 0.279 | 0.000 (0.000–0.228) | 0.989 |
| Gender | Male | 96 | 9 | 0.329 | 0.292 | 0.000 (0.000–0.299) | 0.989 |
| Age | 13–15 | 68 | 6 | 0.333 | 0.298 | 0.000 (0.000–0.390) | 1.000 |
| Age | 16–18 | 132 | 16 | 0.326 | 0.279 | 0.000 (0.000–0.194) | 0.983 |

Supplementary Table S8. High-risk-versus-Rest disparity metrics

| Grouping | Demographic-parity difference | Equal-opportunity difference | False-positive-rate difference | Equalized-odds difference |
| --- | --- | --- | --- | --- |
| Gender | 0.0008 | 0.0000 | 0.0005 | 0.0005 |
| Age group | 0.0152 | 0.0000 | 0.0172 | 0.0172 |

Supplementary Table S9. Strict external-validation feasibility criteria

| Criterion | Required condition | Audit outcome |
| --- | --- | --- |
| Access | Publicly accessible participant-level data | No candidate met all criteria simultaneously |
| Population | Compatible youth-basketball population | Available sources differed in population and/or setting |
| Outcome | Observed, semantically compatible injury-risk outcome | Available outcomes concerned occurrence, severity, rehabilitation, or emergency diagnoses |
| Predictors | Sufficient predictors required by the locked model | Candidate predictor definitions or availability were incompatible |
| Model application | Direct application without relabeling, refitting, or changing the problem definition | Strict external validation not feasible; no nominal validation was manufactured |

Supplementary Table S10. Reconstructed λ implementation audit

| λ | Mean predicted development | Mean predicted High-risk probability | Mean arithmetic utility | Recommendation behavior |
| --- | --- | --- | --- | --- |
| 0 | 22.1261 | 0.131000 | 22.1261 | No change |
| 1 | 22.1261 | 0.131000 | 21.9951 | No change |
| 2 | 22.1261 | 0.131000 | 21.8641 | No change |
| 3 | 22.1261 | 0.131000 | 21.7331 | No change |
| 4 | 22.1261 | 0.131000 | 21.6021 | No change |
| 5 | 22.1261 | 0.131000 | 21.4711 | No change |
| 6 | 22.1261 | 0.131000 | 21.3401 | No change |
| 7 | 22.1261 | 0.131000 | 21.2091 | No change |
| 8 | 22.1261 | 0.131000 | 21.0781 | No change |
| 9 | 22.1261 | 0.131000 | 20.9471 | No change |
| 10 | 22.1261 | 0.131000 | 20.8161 | No change |

The utility decreases linearly by 0.131000 per unit increase in λ. Because predictions and recommendations remain unchanged, this sweep is an arithmetic sensitivity check and not an optimized policy frontier.

Supplementary Table S11. Planned public reproducibility-archive manifest

| Archive component | Purpose | Placement |
| --- | --- | --- |
| Source checksum and target-construction audit | Verify the exact source file and target reconstruction | Aggregate values included here; full outputs in repository |
| Exact split and resampling definitions | Recreate the 800/200 locked split and validation design | Repository file |
| Fold assignments and nested-validation summaries | Audit model selection and variability | Repository files |
| Row-level locked-test predictions | Recompute metrics, curves, confusion matrices, and subgroup summaries | Repository file |
| Subgroup/fairness tables | Audit gender and age failure modes | Aggregate values included here; row-level derivations in repository |
| External-validation candidate-screening log | Document each candidate and exclusion reason | Repository file |
| Model specification and fitted model | Reproduce locked inference | Specification included here; fitted object in repository |
| Figures and figure-supporting outputs | Recreate all four audit figures | Figures in manuscript; source outputs in repository |
| Code and reproducibility reports | Execute the complete computational workflow and verify checksums | Repository files |

## Supplementary Figures

Supplementary Figure S1. Development-score reconstruction coefficients. Points show recovered ordinary least-squares coefficients and horizontal bars show available bootstrap 95% confidence intervals. The intercept estimate was −0.003831; its confidence interval was not available in the embedded outputs.

Supplementary Figure S2. Locked injury-risk metric comparison. The diagnostic all-predictor model is contrasted with the construction-proxy-excluded model across accuracy, balanced accuracy, macro-F1, macro one-vs-rest AUC, High-risk AUC, average precision, sensitivity, and specificity.

Supplementary Figure S3. Locked-test subgroup failure-mode audit. Panel A compares balanced accuracy, macro-F1, and High-risk specificity across gender and age groups. Panel B shows zero High-risk sensitivity with Wilson 95% confidence intervals in every subgroup.

Supplementary Figure S4. High-risk-versus-Rest disparity metrics. Gender and age-group demographic-parity, equal-opportunity, false-positive-rate, and equalized-odds differences are shown. Small values are not evidence of fairness because High-risk sensitivity was zero in every subgroup.

Supplementary Figure S5. Implementation audit of the former λ sweep. Panel A shows the linear arithmetic decline in mean utility. Panels B and C show that predicted development, predicted High-risk probability, and recommendation behavior were invariant across λ = 0–10.

Supplementary Figure S6. Leakage-aware revision and interpretation workflow. The diagram summarizes source auditing, target reconstruction, construction-variable exclusion, locked evaluation, subgroup and calibration diagnostics, implementation auditing, and the resulting bounded interpretation.
